# Comprehensive Profiling Identifies Circulating microRNA Dysregulation in Vietnamese Patients with Heart Failure

**DOI:** 10.3390/ijms26189076

**Published:** 2025-09-18

**Authors:** Bao-Quoc Vu, Phuong Anh Huynh, Nhu Nhat Quynh Nguyen, Niem Van Thanh Vo, Linh Gia Hoang Le, Vu Hoang Vu, Thanh Cong Nguyen, Minh Hoang, Diem My Vu

**Affiliations:** 1 Center for Molecular Biomedicine, University of Medicine and Pharmacy at Ho Chi Minh City, Ho Chi Minh City 70000, Vietnam; vbquoc@ump.edu.vn (B.-Q.V.); huynhanhphuong@ump.edu.vn (P.A.H.); nnqnhu@ump.edu.vn (N.N.Q.N.); thanhniem2611@ump.edu.vn (N.V.T.V.); lghlinh@ump.edu.vn (L.G.H.L.); 2 Department of Internal Medicine, University of Medicine and Pharmacy at Ho Chi Minh City, Ho Chi Minh City 70000, Vietnam; vu.vh@umc.edu.vn; 3 Cardiovascular Center, University Medical Center Ho Chi Minh City, Ho Chi Minh City 70000, Vietnam; thanh.nc@umc.edu.vn; 4 Pasteur Institute of Ho Chi Minh City, Ho Chi Minh City 70000, Vietnam; minhh@pasteurhcm.edu.vn

**Keywords:** circulating miRNAs, heart failure, next generation sequencing

## Abstract

Heart failure (HF) is a complex and multifactorial syndrome with high morbidity and mortality rates worldwide. Accumulative evidence suggests that microRNAs (miRNAs) play critical roles in maintaining cardiac homeostasis. The dysregulation of various miRNAs has been reported in different studies on failing human hearts. However, little is known about their circulatory profile. In this study, comprehensive miRNA profiling was performed by next-generation sequencing for plasma samples of 24 HF and 24 age and sex-matched, non-HF patients. A total of 1391 miRNAs were detected, of which 228 miRNAs and 261 miRNAs were commonly expressed in the HF and non-HF groups, respectively. Eight miRNAs (hsa-let-7b-3p, hsa-miR-92b-5p, hsa-miR-145-3p, hsa-miR-206, hsa-miR-664a-5p, hsa-miR-1307-5p, hsa-miR-1908-5p, and hsa-miR-3074-5p) were found to be dysregulated between HF and non-HF patients. The expression of another seven miRNAs (hsa-miR-589-5p, hsa-miR-30b-5p, hsa-miR-654-3p, hsa-miR-1292-5p, hsa-miR-659-5p, hsa-miR-548d-5p, and hsa-miR-7847-3p) was frequently observed in HF patients but not in non-HF cases. Subsequent analyses of target gene prediction and associated pathways revealed the enrichment of pathways related to vascular development, the cell cycle, and transcriptional activity. These data reveal the expression profile and the dysregulation of circulating miRNAs in our patients with HF.

## 1. Introduction

Heart failure (HF) is a multifactorial syndrome characterized by abnormal changes in heart structure or function that result in insufficient cardiac output [1,2]. HF has been estimated to affect over 64 million people and is one of the most common causes of morbidity and mortality worldwide [3]. Although significant attempts have been made to improve HF therapeutics, its prevalence is projected to rise globally, reflecting population aging and increased numbers of hypertension and diabetes-affected individuals [4,5]. Moreover, HF diagnosis remains a significant challenge because the widely used biomarkers such as natriuretic peptides, while highly sensitive, require improvements in specificity [6]. Hence, an enhanced understanding of HF characteristics at the molecular level will enable the discovery and development of novel HF biomarkers and improve current approaches to disease management.

MicroRNAs (miRNAs) are a group of small (20–25 nucleotides), endogenous, non-coding RNAs that regulate target gene expression at the post-transcriptional level [7,8,9]. In recent years, a large body of evidence has demonstrated the pivotal role of miRNAs in the onset and progression of many cardiovascular diseases, including HF [10,11,12]. The dysregulation of miRNAs has been found in diverse pathological processes of HF, such as cardiac remodeling, hypertrophy, and apoptosis [10]. For instance, hsa-miR-451a was reported to attenuate the expression of high mobility group box 1 (HMGB1) protein, thereby protecting cardiomyocytes under anoxia injury [13]. The role of hsa-miR-122 in regulating cardiovascular inflammation, fibrosis, and oxidative stress by targeting sirtuin 6 (SIRT6), forkhead box O3 (FOXO3), and Apelin has been documented in numerous studies [14,15,16]. A member of the let-7 family, let-7b-5p, was implicated in the development of cardiac remodeling through modulating the TLR7 signaling pathway [17]. MiRNA profiling of failing human hearts has resulted in the identification of unique up and downregulated miRNA subsets [8,18,19]. MiRNAs are expressed in tissues, but many of them can also be detected in the blood. Indeed, circulating miRNAs are highly stable under multiple freeze–thaw cycles, boiling, and long-term storage [20], making them an ideal biomarker for HF diagnosis and prognosis.

Much research on circulating miRNAs in the HF context has investigated the expression of a few reported candidate miRNAs, whereas comprehensive profiling of these remains scarce. It has been suggested that such studies will provide valuable insights into HF pathogenesis that can be employed in diagnosis and to better classify different HF subtypes [21,22]. In the present study, we aimed to define expression profiles of circulating miRNAs in HF and non-HF patients using a next-generation sequencing (NGS) approach and identify differentially expressed (DE) miRNAs and their regulatory signaling pathways.

## 2. Results

### 2.1. Baseline Characteristics of Study Participants

A total of 24 HF and 24 matched, non-HF patients were enrolled in this study. The baseline characteristics of all participants are detailed in Table 1. Recruitment age ranged from 26 years to 86 years for the HF group and from 30 years to 84 years for the non-HF group. For most demographic factors and clinical conditions, no significant difference was observed between the two groups, except for the categories of systolic blood pressure, left ventricular ejection fraction, history of myocardial infarction, coronary syndrome, and dilated cardiomyopathy (Table 1). In contrast, drug usage differed significantly between the HF and non-HF groups (Table 1). NYHA classification and NT-proBNP measurement were only applied to HF patients.

### 2.2. Circulating miRNA Expression Profile

A circulating miRNA expression profile was obtained by NGS performed in all study subjects. The average raw reads were around 2 million per sample. After mapping to known mature miRNA sequences annotated in miRBase, the average mapped reads remained approximately 0.5 million per sample. A total of 1391 miRNAs were detected, of which 1100 miRNAs were obtained from the HF group compared with 1194 miRNAs in the non-HF group. The miRNAs with CPM values less than 1 and that were present in less than 70% of the study population were considered spurious and excluded. A total of 237 miRNAs remained for subsequent analysis (referred to as the common panel, Table S1). The same criteria were applied to the samples in each study group separately, leading to the identification of 228 miRNAs in the HF group and 261 miRNAs in the non-HF group (referred to as specific panels, Table S1).

Regarding miRNA abundance, the top 20 miRNAs with the highest abundance were similar between the two groups. Those miRNAs included hsa-miR-451a, hsa-miR-122-5p, hsa-miR-122b-3p, hsa-miR-148a-3p, hsa-let-7b-5p, hsa-let-7i-5p, hsa-miR-486-5p, hsa-miR-423-5p, hsa-miR-3184-3p, hsa-miR-26a-5p, hsa-let-7a-5p, hsa-miR-99a-5p, hsa-miR-92a-3p, hsa-miR-151a-3p, hsa-miR-126-3p, hsa-miR-25-3p, hsa-let-7f-5p, hsa-let-7g-5p, hsa-miR-486-3p, and hsa-miR-10b-5p. They accounted for 93,4% and 93,5% of the total miRNA abundance in the HF and non-HF groups, respectively. Although the proportion of some miRNAs slightly varied, the top three miRNAs were hsa-miR-451a, hsa-miR-122-5p, and hsa-miR-122b-3p.

**Figure 1:**
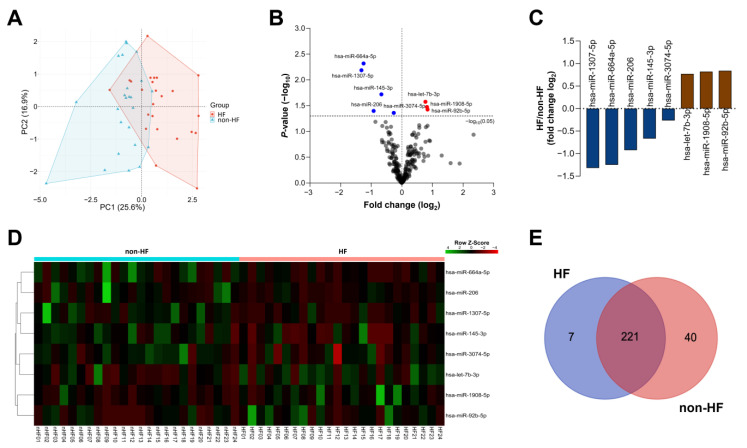
Circulating miRNA profiles in HF and non-HF patients. (**A**) Principal component analysis of DE miRNAs between HF (red) and non-HF (blue) patients. (**B**) Volcano plot displaying the miRNA expression between HF and non-HF patients. Upregulated and downregulated miRNAs are shown in red and blue, respectively. Grey dots indicate miRNAs with no significant change. (**C**) Expression levels of DE miRNAs between HF and non-HF patients. (**D**) Heat map showing the DE miRNA expression in all samples. (**E**) Venn diagram of miRNAs belonging to specific panels of the HF and non-HF groups.

Expression analysis of miRNAs in the common panel revealed eight differently expressed (DE) miRNAs (hsa-let-7b-3p, hsa-miR-92b-5p, hsa-miR-145-3p, hsa-miR-206, hsa-miR-664a-5p, hsa-miR-1307-5p, hsa-miR-1908-5p, and hsa-miR-3074-5p) between HF and non-HF subjects. Of these, principal component analysis showed a prominent cluster of samples from the same group (Figure 1A). A volcano plot was used to visualize the miRNA expression variability between the HF and non-HF groups and the statistical significance (Figure 1B). Among the DE miRNAs, five of them were downregulated, whereas the other three were upregulated significantly in the HF group (Figure 1C). The expression pattern of DE miRNAs is presented in a heatmap for all study participants (Figure 1D). Furthermore, the miRNAs belonging to the specific panel of each group were intersected to define the shared and unique miRNAs between HF and non-HF patients (Figure 1E).

### 2.3. Target Gene Analysis of DE miRNAs Between the HF and Non-HF Groups

**Figure 2:**
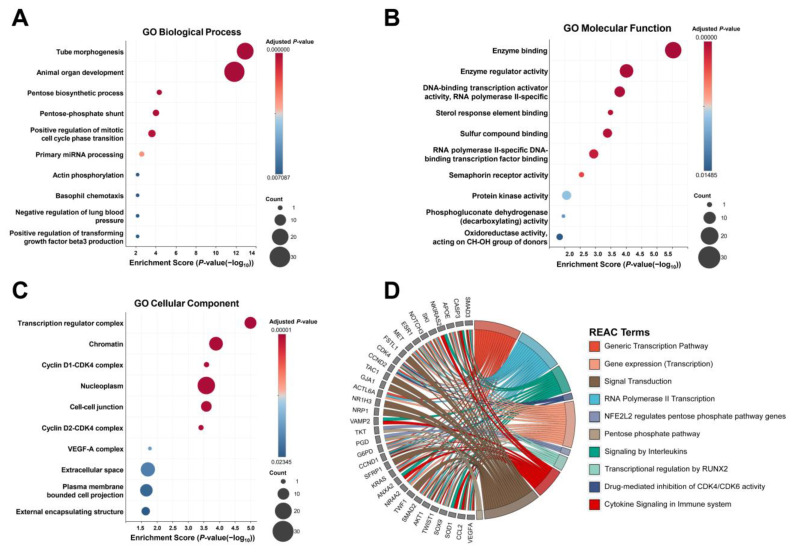
Pathway enrichment analyses for DE miRNAs. (**A**–**C**) Gene Ontology analysis of biological processes, cellular components, and molecular functions for strongly predicted target genes regulated by DE miRNAs. (**D**) Reactome analysis for strongly predicted target genes regulated by DE miRNAs.

We further investigated the biological function of DE miRNAs. Using miRTargetLink 2.0, 42 genes were strongly predicted as regulatory targets of DE miRNAs (Table S2). Subsequent assessment of these genes by Gene Ontology (GO) analysis showed enrichment of diverse pathways related to transcriptional activity, the cell cycle, and vascular formation (Figure 2A–C, Table S3). As GO analysis mainly presents the activities of individual gene products, Reactome (REAC) was also employed to define the physical entities and locations of target genes in their pathways. Their dominant involvement in many pathways related to transcriptional activities was observed (Figure 2D, Table S3).

### 2.4. Target Gene Analysis of Unique miRNAs in HF Patients

**Figure 3:**
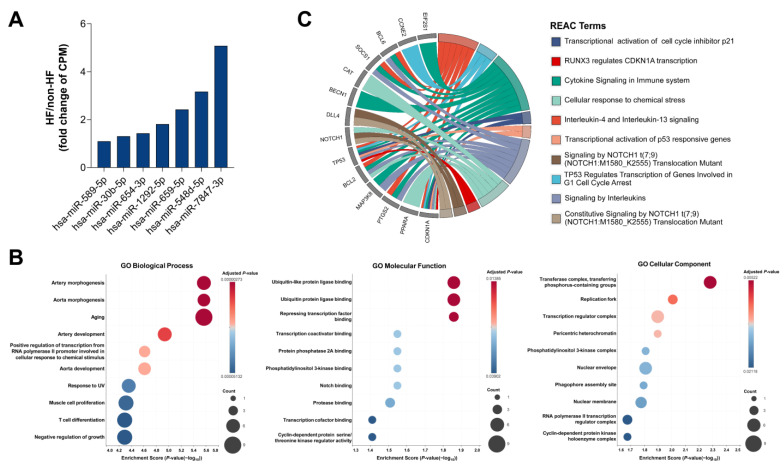
Pathway enrichment analyses for unique miRNAs. (**A**) Expression levels of unique miRNAs between the HF and non-HF groups. (**B**) Gene Ontology analysis of biological processes, cellular components, and molecular functions of strongly predicted target genes regulated by unique miRNAs. (**C**) Reactome analysis for strongly predicted target genes regulated by DE miRNAs.

After the removal of miRNAs with a CPM value less than 1 and that were present in less than 70% of study participants, seven miRNAs (hsa-miR-589-5p, hsa-miR-30b-5p, hsa-miR-654-3p, hsa-miR-1292-5p, hsa-miR-659-5p, hsa-miR-548d-5p, and hsa-miR-7847-3p) were identified in the HF group but not in the non-HF group (Figure 1E, Figure 3A). Subsequent analysis showed that 33 genes were strongly predicted as regulatory targets of these miRNAs (Table S2). Remarkably, the GO analysis results reveal significant enrichment of important pathways correlated to heart formation and development, such as aorta and artery morphology and development, muscle cell proliferation, the Notch pathway, and the Ubiquitin pathway (Figure 3B, Table S4). The REAC analysis also showed that NOTCH1 and RUNX3, two essential factors involved in cardiac cell proliferation and differentiation, were enriched (Figure 3C, Table S4). Collectively, these data indicate that HF might provoke specific cardiac cell responses.

## 3. Discussion

HF remains a significant clinical challenge due to its diverse etiologies and common clinical manifestations that hinder effective diagnosis and treatment. Hence, the investigation of novel biomarkers and pathogenic pathways is of great scientific and clinical importance in HF research. In the present study of 24 HF cases and 24 non-HF patients, comprehensive profiling of circulating miRNAs by NGS revealed distinct miRNA regulation pathways in HF. Based on global miRNome analysis, the expression of eight miRNAs differed significantly between HF and non-HF patients. In addition, the expression of another seven miRNAs was more frequently detected in HF cases. Predicted target gene and pathway analyses of these miRNAs showed the enrichment of essential pathways associated with cardiovascular development and formation, transcriptional activity, and the cell cycle.

In recent years, cumulative evidence has indicated the pivotal roles of miRNAs in heart development and homeostasis [23,24]. Since various cellular functions are regulated by miRNAs, their dysregulation directly involves HF development through multiple processes, including cardiomyocyte apoptosis, cardiac hypertrophy, and myocardial fibrosis [25]. Given that a vast number of miRNAs have been reported in cells, much remains to be discovered about their circulatory profile in order to define them as potential biomarkers and therapeutic targets of HF. Indeed, circulating miRNAs have been examined in many studies on HF patients with different classes of or responses to cardiac therapy [26,27]. Nevertheless, much of the work has concentrated on a few or established panels of circulating miRNAs, leaving some candidates uncovered. Using an unbiased approach, our research exploited NGS to profile circulating miRNAs present in HF patients and non-HF patients. A total of 1391 miRNAs were detected by NGS, of which 237 were considered to be commonly expressed in our population. As some of them have not previously been described in HF, our data have added to the body of knowledge about miRNAs associated with HF.

Among the eight miRNAs with significantly different expression between HF and non-HF patients, circulating hsa-miR-92b-5p and hsa-let-7b-3p have been proposed as candidate biomarkers of and cardiac protectors against HF [28,29,30,31]. The dysregulation of circulating hsa-miR-145-3p, hsa-miR-664a-5p, hsa-miR-1307-5p, hsa-miR-206, and hsa-miR-3074-5p was reported in studies on other cardiovascular diseases, such as myocardial infarction, stroke, coronary syndrome, hypertension, and coronary artery disease [32,33,34,35,36,37]. In a genome-wide study of circulating miRNAs associated with different cardiometabolic phenotypes, some lipid metabolites and lipoproteins were mediated by hsa-miR-1908-5p [38]. In general, our results indicate that changes in the expression pattern of several circulating miRNAs associated with precipitating factors can be observed in HF patients. Analysis of the regulatory networks and pathways formed by predicted targets of DE miRNAs suggested some connections with the cell cycle, vascular formation, and transcriptional activities. Although such data remain scarce in cardiac-derived cells, those connections have been somewhat elucidated in previous research. Particularly, the TFIIB-related factor 2 (BRF2) and SMAD family member 4 (SMAD4) transcription factors were reported as direct targets of hsa-let-7b-3p and hsa-miR-3074-5p, respectively [39,40]. Increased expression of cell cycle genes in myoblasts was observed under miR-664-5p overexpression, whereas hsa-miR-1307-5p markedly reduced the level of MDM4 protein and its downstream Hippo pathway, resulting in cell cycle arrest [41,42]. RAD51, a multifunctional protein playing a central role in DNA replication, was confirmed to be a target of hsa-miR-92b-5p [43]. hsa-miR-1908-5p plays diverse functions in human disorders by modulating PTEN and STAT3 [44]. hsa-miR-145-3p and hsa-miR-206 mediate vascular formation through impacting vascular endothelial growth factor (VEGF) expression [45,46]. Further investigation of DE miRNA cellular function in the cardiovascular context is required in order to strengthen the obtained results.

Intriguingly, certain miRNA alterations were apparent in HF patients but not in non-HF patients. As they are mostly involved in regulatory networks of cardiovascular development and function, aberrant levels of these miRNAs have been documented in vascular diseases, including coronary artery disease and atherosclerosis. Specifically, circulating hsa-miR-589-5p and hsa-miR-1292-5p were differently expressed in coronary artery cases, and hsa-miR-548d-5p level could be implemented to improve risk assessment and treatment response in these cases [31,47,48,49]. The downregulation of serum hsa-miR-30b-5p was noticed in nonresponders to percutaneous coronary intervention [50,51]. A recent study by Hildebrandt’s group reported the more frequent presence of hsa-miR-654-3p in individuals with carotid artery stenosis compared with other types of atherosclerosis [52]. Abnormal expression of plasma hsa-miR-659-5p was proposed to contribute to the development and progression of intracranial aneurysms [53]. Notably, to some extent, our data have partially uncovered hsa-miR-7847-3p’s involvement in HF. The role of these miRNAs in vascular development, muscle cell proliferation, and pathways such as Notch and Ubiquitin has been documented. In particular, hsa-miR-30b-5p was found to control the proliferation and differentiation of vascular smooth muscle cells by mediating muscleblind-like splicing regulator 1 (*MBNL1*) [54]. Peroxisome-proliferator-activated receptor gamma (PPAR-γ), a factor required for normal development of the arterial pole, was reported to be a target of hsa-miR-548d-5p [55,56]. In addition, valvular formation and cardiomyocyte metabolism can be affected by altered expression of hsa-miR-654-3p, hsa-miR-659-5p, hsa-miR-1292-5p, and its target Fibulin 2 (FBLN2) protein [57,58,59]. The ubiquitination proteins tumor necrosis factor receptor-associated factor 6 (TRAF6) and ankyrin repeat and SOCS box containing 12 (ASB12) were found to be the regulatory targets of hsa-miR-589-5p and hsa-miR-7847-3p, respectively [60,61]. Altogether, this information suggests the derailment of vascular-related miRNAs contributing to pathological courses of HF.

## 4. Patients and Methods

### 4.1. Study Design and Participants

**Figure 4:**
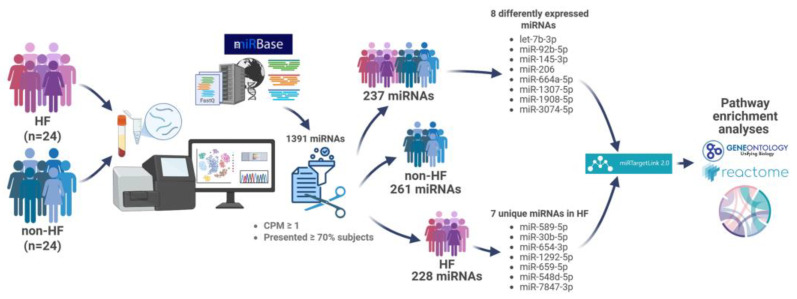
Study flowchart. HF, heart failure; non-HF, non-heart failure; CPM, counts per million.

This cross-sectional study was conducted at the University Medical Center in Ho Chi Minh City, Vietnam, from June 2024 to December 2024. Twenty-four participants were consecutively enrolled for each study group, and convenience sampling was employed (Figure 4). The inclusion criteria for HF cases were as follows: >18 years old; a confirmed diagnosis with stable HF for ≥3 months according to the 2016 Guidelines of the European Society of Cardiology and the HF management protocol issued by the Ministry of Health of Vietnam in 2020; and a New York Heart Association (NYHA) Classification of II–IV, regardless of the cause and left ventricular ejection fraction (LVEF). Non-HF subjects were defined as participants with no known history and symptoms of HF at the time of enrollment and an LVEF ≥ 50% and were matched to HF cases by age (±5 years old) and gender. Participants were excluded if they met one of the following conditions: acute myocardial infarction, surgery or transplantation within three months before enrollment, autoimmune disease, chronic renal failure, known malignancy, pregnancy, or breastfeeding.

### 4.2. Blood Sampling and miRNA Isolation

A total of 4 mL of peripheral blood was collected from each study participant in a Vacutainer EDTA Tube (BD, Franklin Lakes, NJ, USA), left to stand for 30 min at room temperature (RT), and then centrifuged for 15 min at the speed of 4400 rpm. After centrifugation, the plasma was transferred into a new RNase/DNase-free tube and stored at −70 °C until subsequent analyses.

Plasma miRNA was isolated by a miRNeasy Serum/Plasma Advanced Kit (#217204, Qiagen, Hilden, Germany) following the manufacturer’s instructions. miRNA concentration was determined by a Qubit 2.0 Fluorometer with a Qubit microRNA Assay Kit (#Q32880, Invitrogen, Waltham, MA, USA). Isolated miRNAs were stored at −70 °C for subsequent analyses.

### 4.3. miRNA Sequencing and Target Gene Enrichment Analysis

A total of 6.5 μL of isolated miRNA was used for library preparation of small RNAs using the Small RNA Library Prep Set for Illumina (#E7300, NEB, Ipswich, MA, USA). Library quality was assessed by the Tapestation system (Agilent, Santa Clara, CA, USA), and the concentration was measured by the Qubit dsDNA Assay Kit (#Q33230, Invitrogen, Waltham, MA, USA). Libraries were pooled and sequenced on the Miseq (Illumina, San Diego, CA, USA) using the Miseq Reagent kit v3 with 150 cycles (#MS-102-3001, Illumina, San Diego, CA, USA).

Raw sequencing data were demultiplexed to generate a single fastq file for each sample. The sequencing data were analyzed by nf-core/smrnaseq—a bioinformatics best-practice analysis pipeline for small-RNA sequencing. Adapter sequences were trimmed, and all reads shorter than 16 nucleotides were removed by fastp and cutadapt. Trimmed reads were aligned to known mature human miRNAs in miRBase database 22.0 by Bowtie1, while the unaligned reads were filtered by Bowtie2. Post-alignment processing was performed by SAMtools. To compare miRNA expression between samples, miRNA counts were then normalized by counts per million (CPM) using EdgeR. miRNAs with a CPM value less than 1 were removed from further analysis. miRNA target genes were predicted by miRTargetLink 2.0 and subsequently analyzed for pathway enrichment using g:Profiler.

### 4.4. Statistical Analyses

The distribution of continuous variables was determined based on the Shapiro–Wilk test. Continuous variables are presented as mean ± standard deviation if normally distributed or median (interquartile range) if not normally distributed. Categorical variables are expressed as frequencies (percentages). Depending on the distribution, continuous variables were compared by the *t*-test or Mann–Whitney U test. For categorical variables, the differences were assessed by Fisher’s exact test. A *p*-value of <0.05 was considered statistically significant. Statistical analyses were performed using GraphPad Prism 8 (GraphPad Software, La Jolla, CA, USA).

## 5. Limitations

Our study has several limitations. First, an apparent drawback is the small number of participants, as this may introduce an overestimation of some miRNAs while underestimating others whose significance could have been enhanced or blunted in a larger cohort of patients. Second, we only performed a single miRNA profiling analysis for each patient, although the measurements may vary over time. Hence, longitudinal assessment of miRNA expression following disease progression is needed. Third, little information is available for these miRNAs and the signaling pathways related to vascular and cardiac function, regardless of the fact that the main body of evidence suggests that they play a role in activation. Fourth, we cannot exclude the possibility that the miRNA profile might have been influenced by patient comorbidities, disease states, and drug treatments. Therefore, further investigation, including in treatment-naive individuals and healthy controls, is required to consolidate our findings.

## 6. Conclusions

Using a comprehensive approach to profiling circulating miRNAs, we have identified different subsets of miRNAs that appear to be differentially expressed in HF. Analyses of predicted target genes and the regulatory activity of these miRNAs indicated several pathways related to transcriptional activity, cardiovascular formation, and the cell cycle.

## Figures and Tables

**Table 1:** Baseline characteristics of study participants.

Characteristics	Heart Failure Patients (*n* = 24)	Non-Heart Failure Patients (*n* = 24)	*p*-Value
Gender, (*n*; % Male)	12; 50.0	12; 50.0	1.000
Age (years)	55.0 ± 15.7	54.3 ± 14.6	0.865
Body mass index (kg/m^2^)	24.6 ± 3.8	24.5 ± 3.8	0.904
Heart rate (beats/minute)	77.0 ± 12.1	83.9 ± 12.7	0.060
Systolic blood pressure (mm/Hg)	120.5 ± 18.5	131.1 ± 13.5	0.028 *
Diastolic blood pressure (mm/Hg) ^a^	70.0 ± 15.0	79.0 ± 10.0	0.318 ^b^
NYHA II (*n*; %)	16; 66.7	0	-
NYHA III (*n*; %)	7; 29.2	0	-
NYHA IV (*n*; %)	1; 4.2	0	-
% Left ventricular ejection fraction	38.7 ± 13.7	68.7 ± 7.09	<0.0001 *
Hypertension (*n*; %)	21; 87.5	18; 75.0	0.461
Diabetes (*n*; %)	8; 33.3	5; 20.8	0.517
Hyperlipidemia (*n*; %)	16; 64.0	9; 37.5	0.089
History of myocardial infarction (*n*; %)	9; 36.0	0; 0.0	0.002 *
Myocarditis (*n*; %)	1; 4.2	1; 4.2	1.000
Ischemic heart disease (*n*; %)	4; 16.0	1; 4.2	0.349
Coronary syndrome (*n*; %)	11; 45.8	0; 0.0	0.0002 *
Angina pectoris (*n*; %)	1; 4.2	0; 0.0	>0.999
Dilated cardiomyopathy (*n*; %)	8; 33.3	0; 0.0	0.004 *
Angiotensin receptor blocker (*n*; %)	20; 83.3	12; 50.0	0.031 *
Betablocker (*n*; %)	21; 87.5	4; 16.0	<0.0001 *
Statin (*n*; %)	18; 75.0	10; 41.7	0.039 *
Aspirin (*n*; %)	5; 20.8	0; 0.0	0.049 *
Clopidogrel (*n*; %)	11; 45.8	1; 4.2	0.002 *
Calcium channel blocker (*n*; %)	1; 4.2	7; 29.2	0.048 *
NT-proBNP (pg/mL) ^a^	1806 (542–3201)	n/a	-

* Statistical significance at *p*-values < 0.05; ^a^ Median (IQR); ^b^ Mann–Whitney U test; n/a, not available.

## Data Availability

The data presented in this study are available on request from the corresponding author.

## Supplementary Materials

The following supporting information can be downloaded at: https://www.mdpi.com/article/10.3390/ijms26189076/s1.
